# Maize intercropping enriches plant growth-promoting rhizobacteria and promotes both the growth and volatile oil concentration of *Atractylodes lancea*

**DOI:** 10.3389/fpls.2022.1029722

**Published:** 2022-10-24

**Authors:** Zheng Peng, Xiuzhi Guo, ZengXu Xiang, Dahui Liu, Kun Yu, Kai Sun, Binbin Yan, Sheng Wang, Chuanzhi Kang, Yang Xu, Hongyang Wang, Tielin Wang, Chaogeng Lyu, Wenjun Xue, Li Feng, Lanping Guo, Yan Zhang, Luqi Huang

**Affiliations:** ^1^ State Key Laboratory and Breeding Base of Dao-di Herbs, Resource Center of Chinese Materia Medica China Academy of Chinese Medical Sciences, Beijing, China; ^2^ Institute of Traditional Chinese Medicine Health Industry, China Academy of Chinese Medical Sciences, Nanchang, China; ^3^ College of Horticulture, Nanjing Agricultural University, Nanjing, China; ^4^ College of Pharmacy, Hubei University of Chinese Medicine, Wuhan, China; ^5^ Nanjing WaMing Agricultural Technology Co., Ltd., Nanjing, China

**Keywords:** *Atractylodes lancea* (Thunb.) DC., intercropping, root barrier, volatile oil, rhizosphere, soil physicochemical properties, PGPR, maize

## Abstract

In the *Atractylodes lancea* (*A. lancea*)-maize intercropping system, maize can promote the growth of *A. lancea*, but it is unclear whether this constitutes an aboveground or belowground process. In this study, we investigated the mechanisms of the root system interaction between *A. lancea* and maize using three different barrier conditions: no barrier (AI), nylon barrier (AN), and plastic barrier (AP) systems. The biomass, volatile oil concentration, physicochemical properties of the soil, and rhizosphere microorganisms of the *A. lancea* plant were determined. The results showed that (1) the *A. lancea* - maize intercropping system could promote the growth of *A. lancea* and its accumulation of volatile oils; (2) a comparison of the CK, AI, and AP treatments revealed that it was the above-ground effect of maize specifically that promoted the accumulation of both atractylon and atractylodin within the volatile oils of *A. lancea*, but inhibited the accumulation of hinesol and β-eudesmol; (3) in comparing the soil physicochemical properties of each treatment group, intercropping maize acidified the root soil of *A. lancea*, changed its root soil physicochemical properties, and increased the abundance of the acidic rhizosphere microbes of *A. lancea* at the phylum level; (4) in an analysis of rhizosphere microbial communities of *A. lancea* under different barrier systems, intercropping was found to promote plant growth-promoting rhizobacteria (PGPR) enrichment, including *Streptomyces*, *Bradyrhizobium*, *Candidatus Solibacter*, *Gemmatirosa*, and *Pseudolabrys*, and the biomass of *A. lancea* was significantly influenced by PGPR. In summary, we found that the rhizosphere soil of *A. lancea* was acidified in intercropping with maize, causing the accumulation of PGPR, which was beneficial to the growth of *A. lancea*.

## Introduction

*Attractylodes lancea* (*A. lancea*) (Chinese: Cangzhu) is used in traditional Chinese medicine (TCM) and has become valued in recent years for its high medicinal and economic value. The rhizome of this plant has been used in clinics to treat rheumatic diseases, digestive problems, night blindness, and influenza (Wang et al., 2008; Nie et al., 2018). *A. lancea* is a perennial plant that is typically cultivated in long-term continuous monocultures. This practice, however, makes the plant prone to soil-borne diseases and continuous crop obstacles, which negatively affect the yield and quality of *A. lancea* (Wang et al., 2016; Chen et al., 2021). Many other medicinal materials and crops suffer from this same issue (Wang, 2020; Gu et al., 2021; Ding et al., 2022). The monoculture cultivation problem has become one of the main challenges hindering sustainable medicinal herb production.

By increasing plant diversity, intercropping serves as an important strategy for restoring the microecological balance of the soil and achieving sustainable agricultural development (Li, 2016). In previous studies, diverse intercropping patterns were found to improve the soil’s micro-ecological environment and increase plant productivity (Raseduzzaman, 2016). Researchers have recently revealed that belowground interactions contribute to plant productivity more than aboveground interactions do, and such interactions involve both the microbial community and physicochemical properties of the soil (Fusuo and Long, 2003; Walker et al., 2009; Bai et al., 2022b). The root microbiota, regarded as the second genome of the plant, promotes the growth, development, and quality of Chinese herbal medicines by influencing their absorption of nutrients and resistance against both biotic and abiotic stresses (Vandenkoornhuyse et al., 2015; Martin et al., 2017; Bai et al., 2022a). Research has shown diversified cropping systems to result in higher soil microbial abundance and diversity, as such systems act by altering the dominant soil microbial taxa and communities (Li et al., 2020; Tian et al., 2020; Lin et al., 2022).

Numerous studies have shown that maize can act as a fitting intercropping partner for many medicinal plants and crops (Li, 2020; Liu et al., 2021; Tripathi et al., 2021). Maize promotes the sustainable productivity of intercropped plants by increasing beneficial soil microorganisms, changing the microbial structure, increasing the microbial abundance, suppressing the occurrence of diseases, and promoting nitrogen uptake (Fan et al., 2019; Chang et al., 2020; Huang et al., 2022). In a previous study, maize was selected among various crops for its superior yield and quality advantage conferred to *A. lancea*, thereby revealing itself as a well-matched intercropping crop. However, the mechanism of the intercropping advantage brought by maize to *A. lancea* has not yet been elucidated.

In this study, by testing the three respective conditions of using no barrier, plastic, and nylon root barriers in the *A. lancea* - maize root, we examined the influence of maize on the yield and quality of *A. lancea* from the perspective of the soil rhizosphere microbiome and physicochemical properties of the soil. This study intends to determine: (1) the effect of maize intercropping on the rhizosphere microbiome structure and abundance in *A. lancea*-cultivated soil; (2) the effect of maize intercropping on the physicochemical properties of *A. lancea*-cultivated soil; and (3) the relationship between the growth and development, volatile oil content, soil rhizosphere microbiome community, and soil physicochemical properties of *A. lancea*.

## Materials and methods

### Experimental site

Field experiments were conducted in the Lishui District, Nanjing City (119°6′38″E, 31°36′2″N, altitude 40 m) in November 2019 during a north subtropical monsoon climate. The area is characterized as having an annual average temperature of 16.0°C, annual average relative humidity of 77%, annual average precipitation of 1147.0 mm, annual average rainy days of 124 days, annual average sunshine hours of 1969.0 hours, and annual average frost-free period of 224 days. All experimental sites were newly cultivated (**Table 1**), and random block experiments were used.

**Table 1:** Physicochemical properties of soil samples from experimental sites (n ≥ 5).

	TN (mg/kg)	TP (mg/kg)	TK (g/kg)	TOC (g/kg)	${\text{NH}}_{4}^{+}$ (mg/kg)	Av. P (mg/kg)	Av. K (mg/kg)	pH
Experimental site	850.68	308.00	11.93	7.39	25.55	5.70	84.60	5.01

TN, total nitrogen; TP, total phosphorus; TK, total kalium; TOC, total organic carbon; ${\text{NH}}_{4}^{+}$ , ammonium nitrogen; Av.P, available phosphorus; Av.K, available kalium.

### Experimental design and field management

**Figure 1:**
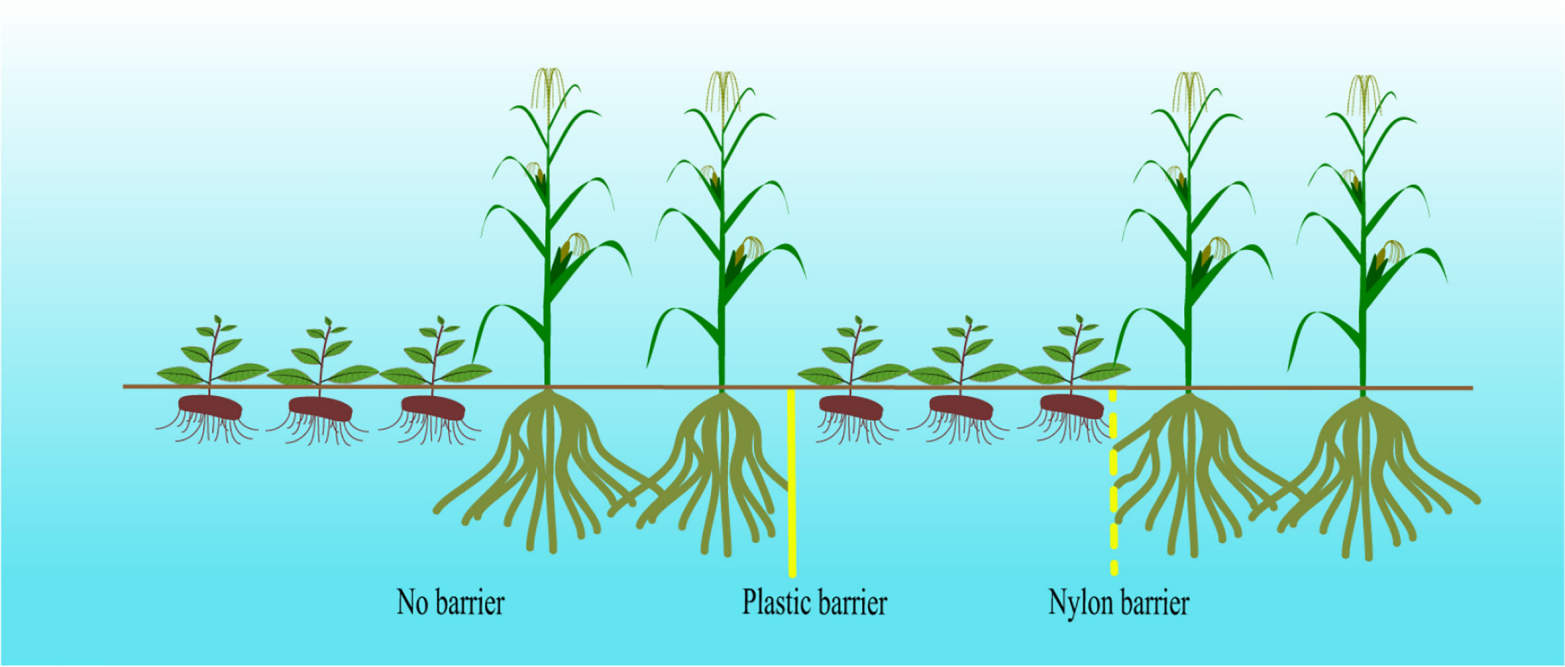
Different root barriers separating *A. lancea - maize* intercropping.

The intercropping experiment was divided into 4 treatments with 4 replicates each: (1) *A. lancea* grown separately as monocultures (CK); (2) *A. lancea* - maize intercropping without a root barrier (AI); (3) *A. lancea* - maize intercropping separated by a nylon barrier (AN), which prohibited the roots from intermingling between species while permitting the exchange of root exudates, water, and nutrients; (4) *A. lancea* - maize intercropping separated by a plastic barrier (AP) to completely eliminate the underground effect of the intercropping system (**Figure 1**). Select *A. lancea* seedlings with similar growth conditions, good resulting growth, and no disease were transplanted into the field and covered with straw in November 2019. The row spacing of *A. lancea* was 30 × 20 cm. The maize was planted in April of the second year (April 2020) with a row spacing of 30×40 cm. Each planting site was fertilized with 100 grams of compound fertilizer (N+P_2_O_5_+K_2_O 5%, organic matter 45%) after the *A. lancea* or maize was planted. The experiment used a randomized complete block design with four replicates, and each experimental plot was 10 m^2^ (2 m × 5 m), and as shown in **Figure 1**, two rows of maize were planted after three rows of *A.lancea*, and the planting ratio was 3: 2.

### Sample collection and measurement of biomass

*A. lancea* and its accompanying rhizosphere soil was collected in July 2020. The *A. lancea* plant roots were carefully removed from the soil and shaken by hand to remove loosely attached soil (not rhizosphere soil). Then, soils tightly adhering to roots were removed by a sterile brush (rhizosphere soils). And these fresh rhizosphere soil samples were stored in dry ice at −80°C for DNA extraction. Six *A. lancea* plants were randomly selected from each experimental plot for biomass and yield analysis, including both their above- and belowground parts. Measurements included plant height, branch number, sprout number, fibrous root, stem diameter, fresh weight, and rhizome weight.

### Determination of volatile oil content

The rhizome of *A. lancea*. were collected and dried in a 40°C oven for one week to constant weight, then crushed it to a<0.3 mm size. The drying of *A. lancea* as well as the extraction and determination of volatile oils were conducted according to Peng et al. (2021). The method is accurate, fast, and reproducible.

### Determination of soil physicochemical properties

To measure soil total nitrogen (TN), total phosphorus (TP), and total kalium (TK), the soil samples were dried indoors by airflow, cleaned by removing the fine roots, and passed through a 0.25 mm soil sieve (Wang et al., 2022). The total organic carbon (TOC) was measured using the kalium dichromate external heating method. The soil pH was measured using the potential method. The contents of ammonium nitrogen ( ${\text{NH}}_{4}^{+}$ ), available phosphorus (Av.P), and available kalium (Av.K) were measured using assay kits manufactured by Sinobestbio Technology Co., Ltd., (Shanghai, China) according to the manufacturer’s instructions.

### DNA extraction, library construction, and metagenomic sequencing

Total genomic DNA was extracted from *A. lancea* rhizosphere soil samples using the E.Z.N.A.^®^ Soil DNA Kit (Omega Bio-tek, Norcross, GA, U.S.) according to the manufacturer’s instructions. The concentration and purity of the extracted DNA were determined by TBS-380 and NanoDrop2000, respectively. The quality of the DNA extracts was checked by conducting electrophoresis on 1% agarose gels. DNA extracts were fragmented to an average size of ~400 bp using Covaris M220 (Gene Company Limited, China) for paired-end library construction. The paired-end library was constructed using NEXTFLEX Rapid DNA-Seq (Bioo Scientific, Austin, TX, USA). Adapters containing the full complement of sequencing primer hybridization sites were ligated to the blunt end of the fragments. Paired-end sequencing was performed on the Illumina NovaSeq/Hiseq Xten system (Illumina Inc., San Diego, CA, USA) at Majorbio Bio-Pharm Technology Co., Ltd. (Shanghai, China) using NovaSeq Reagent Kits/HiSeq X Reagent Kits according to the manufacturer’s instructions (www.illumina.com) (Yang et al., 2022).

### Sequence quality control and genome assembly

The data were analyzed on the free online platform Majorbio Cloud Platform (www.majorbio.com). The paired-end Illumina reads were trimmed of their adaptors, and low-quality reads (length<50 bp or with a quality value<20 or having N bases) were removed by fastp (Chen et al., 2018) (https://github.com/OpenGene/fastp, version 0.20.0).

### Gene prediction, taxonomy, and functional annotation

Open reading frames (ORFs) from each assembled contig were predicted using MetaGene (Noguchi et al., 2006) (http://metagene.cb.k.u-tokyo.ac.jp/). The predicted ORFs with lengths equal to or exceeding 100 bp were retrieved and translated into amino acid sequences using the NCBI translation table, which is available online (http://www.ncbi.nlm.nih.gov/Taxonomy/taxonomyhome.html/index.cgi?chapter=tgencodes#SG1). A non-redundant gene catalog was constructed using CD-HIT (Fu et al., 2012) (http://www.bioinformatics.org/cd-hit/, version 4.6.1) with 90% sequence identity and 90% coverage. After quality control, the resulting reads were mapped to the non-redundant gene catalog with 95% identity using SOAPaligner (Li et al., 2008) (http://soap.genomics.org.cn/, version 2.21), and the gene abundances in each sample were evaluated. The amino acid sequences of the non-redundant gene catalog were aligned to the NCBI NR database with an e-value cutoff of 1e^-5^ using Diamond (Buchfink et al., 2015) (http://www.diamondsearch.org/index.php, version 0.8.35) for taxonomic annotations.

### Statistical analysis

Microsoft Excel 2016 and SPSS v26.0 (SPSS Inc., Chicago, United States) were used for statistical and correlation analyses. The results were expressed as means ± standard deviations (S.D.). One–way analysis of variance (ANOVA) followed by the Fisher Protected Least Significance Difference (LSD) test was performed to determine the main effects. The figures in the manuscript were created with Adobe Illustrator CS6 and GraphPad Prism 8.

## Results

### Intercropping promoted biomass accumulation in *A. lancea*

**Figure 2:**
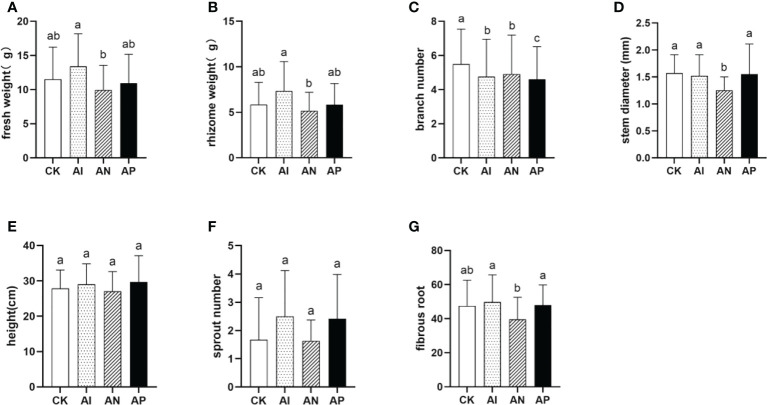
The effect of different root intercropping treatments imposed on *A.lancea-maize* on the growth and development of *A. lancea.* **(A)** fresh weight, **(B)** rhizome weight, **(C)** branch number, **(D)** stem diameter, **(E)** plant height, **(F)** sprout number, **(G)** fibrous root. Data are shown as means ± SD. A-G: n=24. Lower-case letters represent significant differences (one-way ANOVA, *P*<0.05).

To assess the advantages of *A. lancea - maize* intercropping in promoting the growth and development of *A. lancea*, seven agronomic traits of *A. lancea* were examined (**Figure 2**). The AI, AN, and AP treatments were distinguished by using three different root barriers in *A. lancea - maize* intercropping, thereby respectively representing root system interactions completely, partially, or not at all. In comparison with the CK treatment, the fresh weight and rhizome weight were increased by 16.5% and 25.9% under the AI treatment (**Figures 2A, B**), but branch number was markedly decreased by 13.4% (**Figure 2C**). Other agronomic traits saw no significant change between the AI and CK treatments (**Figures 2D–G**). These results indicate that maize intercropping promotes biomass accumulation in *A. lancea*. With declining interactions in the root systems employed by respective conditions, both the fresh weight and rhizome weight correspondingly showed decreasing trends under both the AN and AP treatments as compared with the AI treatment. The results of the agronomic traits analysis showed that the root system interactions of *A. lancea - maize* are involved in the formation of the intercropping advantage and promoted the accumulation of both the fresh weight and rhizome weight.

### Intercropping affected the proportional relationship of four volatile oils in *A. lancea* rhizomes

**Figure 3:**
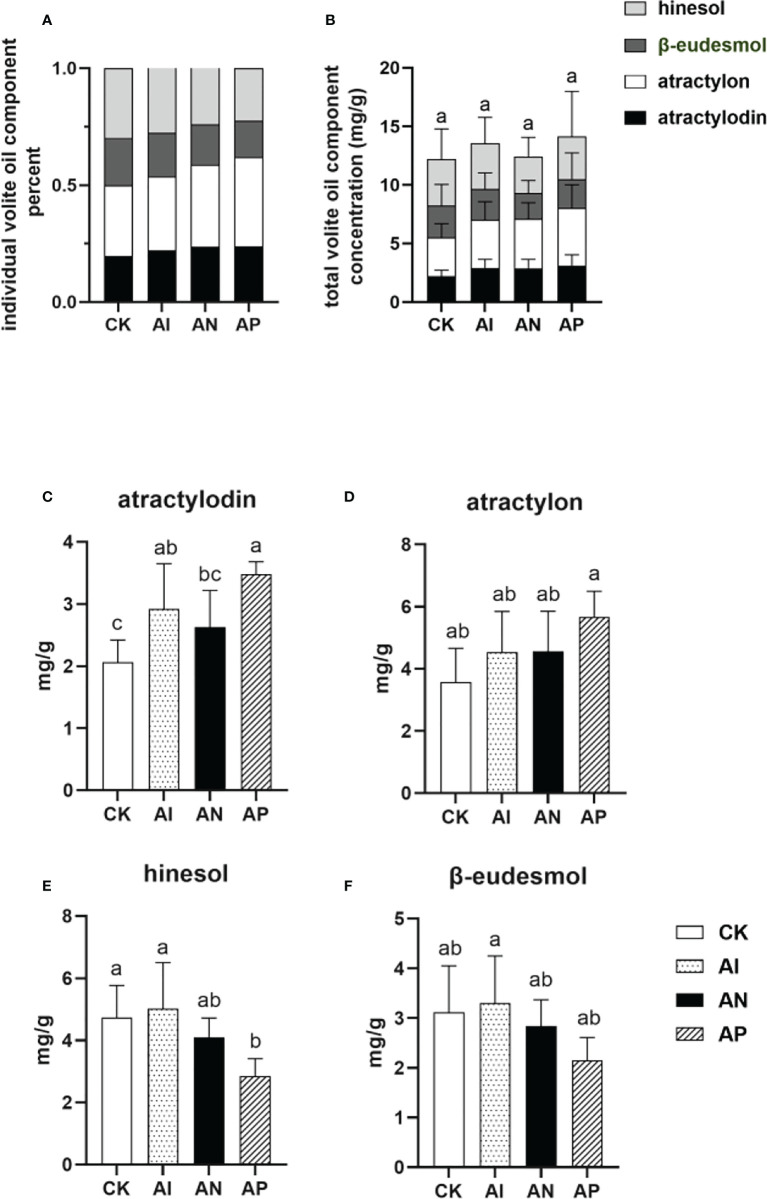
The concentrations of four volatile oils in *A. lancea* rhizomes under different root intercropping treatments in *A. lancea - maize.* **(A)** the proportion of individual components in the four volatile oils, **(B)** individual volatile oil component concentration, **(C)** atractylodin, **(D)** atractylon, **(E)** hinesol, **(F)** β-eudesmol. Data are shown as means ± SD. A-D: n=6. Lower-case letters represent significant differences (one-way ANOVA, *P*<0.05).

The proportion of the individual components comprising the volatile oils of *A. lancea* is an important characteristic for determining the quality of the plant. Rhizomes of the best quality (termed ‘Dao-di’ (Geo-authentic)) possess a higher proportion of both atractylon and atractylodin compared to hinesol and β-eudesmol (Guo et al., 2002). As shown in **Figures 3A, B**, the proportion of the concentrations of atractylon and atractylodin to the total volatile oil concentration in the AI treatment was higher than that of the CK treatment (**Figure 3A**). However, the total combined amount of the four volatile oils saw no significant differences between conditions either with or without underground root system interactions (**Figure 3B**), though the atractylodin concentration was significantly higher in the AI treatment than the CK treatment (**Figure 3C**). The atractylon concentration was slightly higher in AI treatment compared to the CK treatment (**Figure 3D**). The hinesol and β-eudesmol concentrations were no different between the AI and CK treatments (**Figures 3E, F**). These results showed that the quality of *A. lancea* was promoted by intercropping it with maize compared to subjecting it to the CK treatment. With an increase in the root system interactions corresponding to the treatment order of AP< AN< AI, the concentrations of hinesol and β-eudesmol were observed to be improved accordingly (**Figures 3E, F**), while the atractylon content declined (**Figure 3D**). Although lacking a similar accumulation trend with atractylon, atractylodin was higher in the AP treatment than in the AI treatment, which was consistent with the results for atractylon (**Figure 3D**). In summary, *A. lancea* - maize intercropping improved the quality of *A. lancea*, and root system interactions favored the accumulation of hinesol and β-eudesmol but inhibited the accumulation of atractylon.

### Effect of intercropping on the physicochemical properties of *A. lancea*

**Figure 4:**
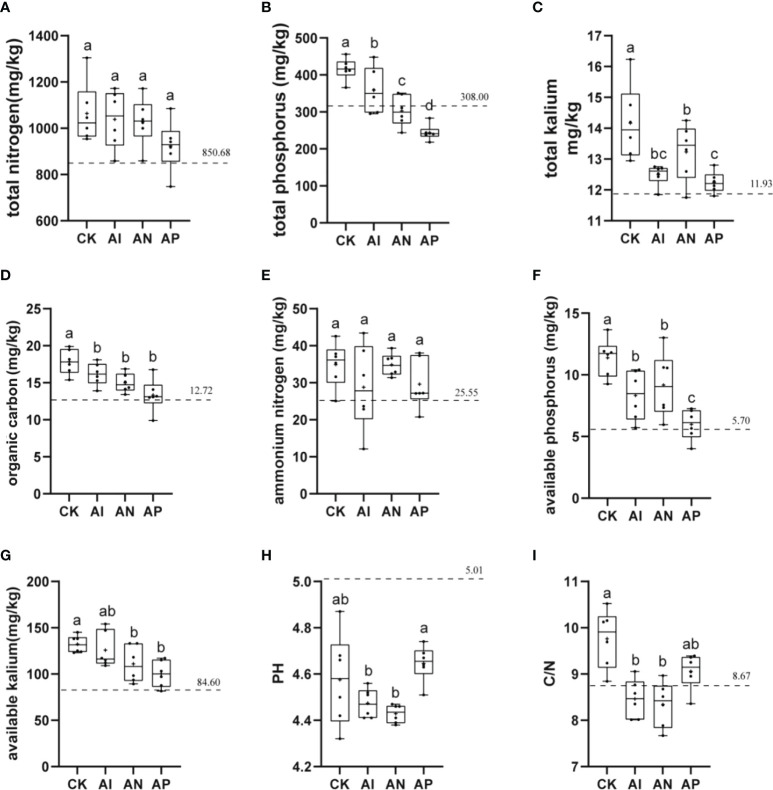
The effects of different root system intercropping treatments on the soil physicochemical properties of *A. lancea* in *A. lancea - maize* intercropping. **(A)** total nitrogen (TN), **(B)** total phosphorus (TP), **(C)** total kalium (TK), **(D)** total organic carbon (TOC), **(E)** ammonium nitrogen ( ${\text{NH}}_{4}^{+}$ ), **(F)** available phosphorus (Av.P), **(G)** available kalium (Av.K), **(H)** pH, **(I)** C/N ratio. Data are shown as means ± SD. **(A-I)**: n=6. Lower-case letters represent significant differences (one-way ANOVA, *P*<0.05).

The physicochemical properties of soil, which are closely related to plant growth, were examined to analyze the effect of maize intercropping on the physicochemical properties of *A. lancea*. The basic physicochemical properties of the bulk soil from the experimental sites are represented by a dotted line in each graph. The rhizosphere soil pH of *A. lancea* was lower than that of the bulk soil. The *A. lancea* root system has the ability to acidify the surrounding soil. There was no clear significant difference observed in the soil pH between the AP and CK treatments (**Figure 4H**). However, the soil pH of the AI and AN treatments were significantly lower than that of the CK treatment. These results suggest that the maize root system had enhanced rhizosphere acidification compared to *A. lancea*. Similar results were obtained for soil carbon levels regarding the nitrogen ratio (**Figure 4I**). As compared with the CK treatment, the TOC, TP, TK, Av.P, and Av.K of *A. lancea* soil was decreased by 9.37%, 13.75%, 11.85%, 26.87%, and 4.92% in the AI treatment (**Figure 4**). With the enhancement in the root interactions of *A. lancea - maize* corresponding to the order of AP< AN< AI, the contents of TOC, TP, and Av.K were found to be improved. The contents of TN and ${\text{NH}}_{4}^{+}$ exhibited no significant differences among all the groups (**Figures 4A, E**). These preliminary results demonstrate that TOC, TP, and Av.K of the *A. lancea* rhizosphere is affected by the root system interactions under *A. lancea - maize* intercropping.

### Intercropping effects on the rhizosphere microbial community structure of *A. lancea*

**Figure 5:**
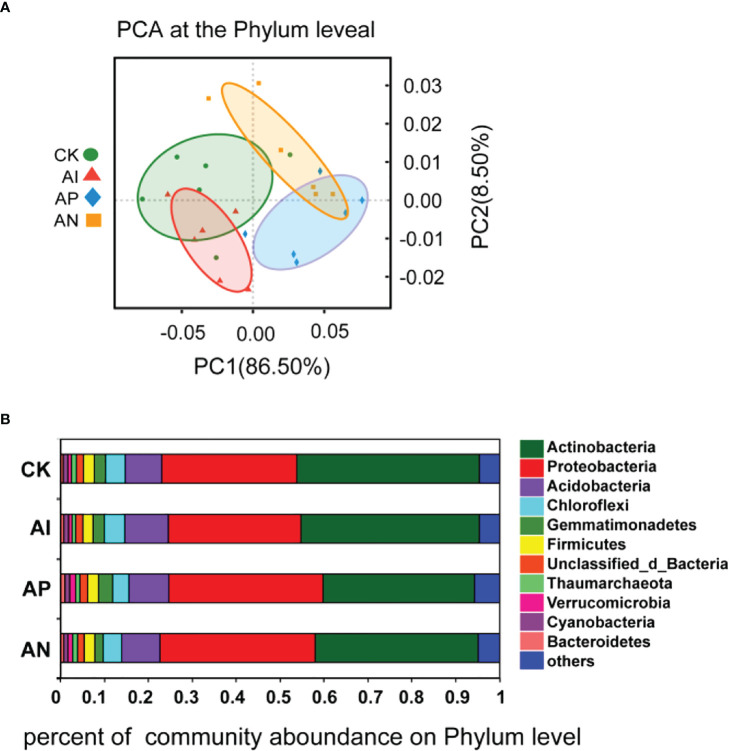
PCA analysis of the microbial community structure **(A)** and the microbial community composition **(B)** at the phylum level.

To further understand the effect of maize intercropping on the growth and quality of *A. lancea*, we performed metagenomic assays on the rhizosphere microbial community of *A. lancea*. PCA analysis at the phylum level was performed on the microbial communities obtained from the different treatment groups, and the rhizosphere microbial communities of the four treatments were found to be different due to different intercropping treatments (**Figure 5A**). It can be seen from **Figure 5B** that the rhizosphere microbial community composition of *A. lancea* did not change across treatment groups, but its microbial abundance changed significantly. At the phylum level, the act of intercropping *A. lancea* with maize significantly changed the abundance of microbes in the rhizosphere microbial community of *A. lancea*. The top 2 phyla in order of their abundance were *Actinobacteria* (relative abundance ≥ 30%) and *Proteobacteria* (relative abundance ≥ 20%), followed by *Acidobacteria*, *Chloroflexi*, and *Gemmatimonadetes* (**Figure 5B**). Observing the rhizosphere flora of *A. lancea* across the different treatments reveals that intercropping significantly affected the abundance of *Actinobacteria* and *Proteobacteria*, especially in the AN and AP treatment compared to the CK treatment, as well as the AN treatment and AP treatment compared to the AI treatment (**Figures S1A–D**). There was no significant difference observed in the abundance of *Actinobacteria* or *Proteobacteria* between the CK treatment and AI treatment (**Figure S1E**). There was also no significant difference observed between the AN and AP treatment (**Figure S1F**).

### Effects of intercropping on the microbial community abundance of the *A. lancea* rhizosphere

**Figure 6:**
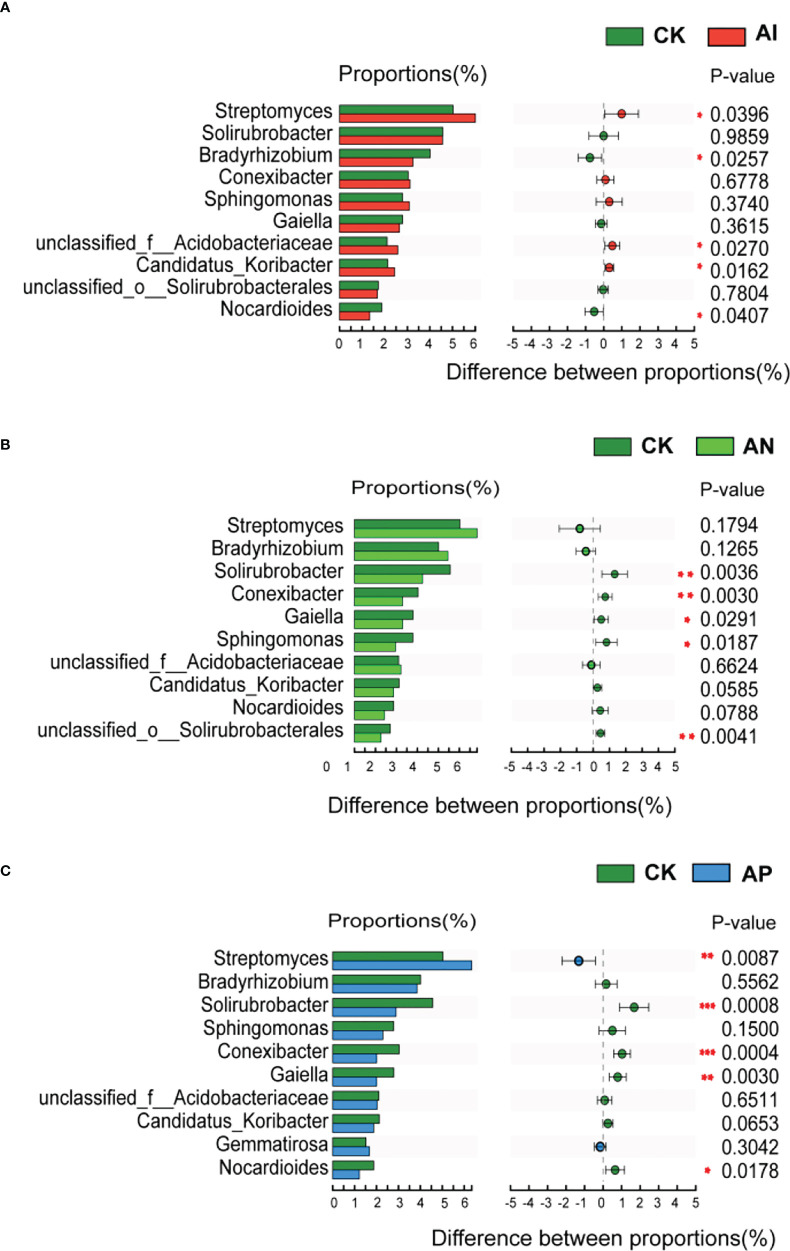
Abundance comparison of the top 10 genera comprising the rhizosphere microbial community in *A. lancea* between the CK treatment and other different root intercropping treatments in *A. lancea* - maize intercropping. Comparison of microbial abundance at the genus level between the **(A)** CK and AI treatment, **(B)** CK and AN treatment, **(C)** CK and AP treatment. (*P<0.05; **P<0.01; ***P<0.001).

At the genus level, **Figure 6** shows that the relative abundance of the *A. lancea* rhizosphere microbial community significantly differed between the CK treatment and the other intercropping treatments. There were 5 genera observed under the AI treatment, 5 under the AP treatment, and 5 under the AN treatment in the top 10 genera compared with the CK treatment, respectively (**Figure 6**). The AI treatment significantly promoted the abundance of *Streptomyce*s, and *unclassified_o:Acidobacteriaceae*, *Candidatus_Koribacter*, while having significantly inhibited *Bradyrhizobium* and *Nocardioides* (**Figure 6A**). *Solirubrobacter*, *Conexibacter*, *Gaiella*, *Sphingomonas*, and *unclassified_o:Solirubrobacterales* were significantly inhibited under the AN treatment compared with the CK treatment (**Figure 6B**). Comparison between the AP and CK treatment (**Figure 6C**) showed that *Streptomyces* and *Gemmatimonas* were significantly promoted, while *Solirubrobacter*, *Conexibacter* and *Gaiella* were significantly inhibited. *Bradyrhizobium* were found to be significantly promoted in the AN treatment compared with the AI treatment, as well as in the AP treatment compared with the AI treatment, while *Solirubrobacter*, *Conexibacter*, *Sphingomonas*, and *Candidatus_koribacter* were inhibited (**Figure S2**).

### Correlation analysis among the rhizosphere microorganisms, physicochemical properties of the soil, volatile oil content, and biomass of *A. lancea*

**Figure 7:**
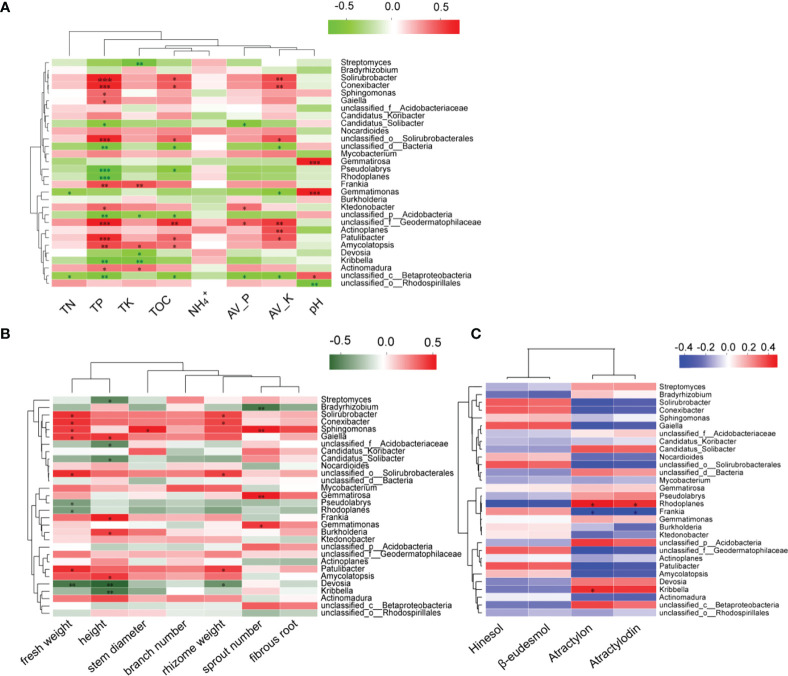
Spearman correlation analysis of the top 30 represented microbes at the genus level against the **(A)** soil physicochemical properties, **(B)** growth and development, and **(C)** four volatile oils of *A. lancea.* (*P<0.05; **P<0.01; ***P<0.001).

In order to explore the relationship between the microbial community and the growth of *A. lancea*, its volatile oil content, and soil physicochemical properties (**Figure 7**), Spearman correlation analysis was performed. Results show that the soil TP, TOC, Av.P, and Av.K were closely related to the microorganisms comprising the *A. lancea* rhizosphere. The pH was negatively correlated with microbial abundance at the genus level, while the TN, TP, TK, TOC, Av.P, and Av.K were positively correlated with most microbial abundance (**Figure 7A**). Likewise, microbes were found to be strongly associated with the growth and development of *A. lancea* especially in relation to its roots (**Figure 7B**) while only few microorganisms are related with atractylon and atractylodin of four volatile oils of *A. lancea.* (**Figure 7C**). The physicochemical properties of the soil were determined to be weakly correlated with the growth and quality of *A. lancea* rhizoma (**Table S1**), but showed a strong correlation with the resident microorganisms, especially for the soil concentration changes of the total phosphorus, organic carbon, available phosphorus, and available kalium (**Figure 7A**).

## Discussion

### Increased yield and volatile oil concentration of *A. lancea* by maize intercropping without root barriers

Much research has shown that improving biodiversity can increase the productivity of entire plant systems (Brooker et al., 2015; Wang et al., 2018; Cong, 2020). There are two predominant mechanisms underlying this effect in intercropping, which are (i) resource complementarity and (ii), niche partitioning (Yu et al., 2021). In our previous research, we found that the intercropping of *A. lancea* with maize, calendula, marigold, etc. increased the fresh weight and the rhizome weight of *A. lancea* (Peng et al., 2021). Similar results have been found in the current study, although the fresh weight and rhizome weight of *A. lancea* under the AI treatment was not significantly higher than that under the CK treatment. This inconspicuous effect of intercropping may be due to the fact that our study provided adequate nutrition for *A. lancea*, and this must be considered against the fact that crop diversity largely exerts its effects by alleviating a stressful environment for plants to increase their growth; for example, in combating nutrient stress (Liancourt and Dolezal, 2021). In one study, the plant-plant stimulatory effect was observably decreased with the reduction of stress in the intercropping of maize and grass bean under phosphorus and water deficiency conditions (Zhu et al., 2022). Similarly, under either water sufficiency or drought stress conditions, intercropping increased grain yield by 14% and 93%, respectively, over monocropping (Willey, 1990).

In many plant-plant interaction studies, the strength of the rhizosphere interaction determines plant growth outcomes (Zhang et al., 2017; Xiao et al., 2020; Li et al., 2021), but in our study, some rhizosphere interactions of maize evidently failed to promote the growth of *A. lancea*. Maize is more competitive than *A. lancea* due to its dominant position in the nylon barrier intercropping system (Li et al., 2011; Worku, 2014; Zhu et al., 2022), and is hence better able to absorb nutrients and soil moisture through the nylon barrier. Therefore, the rhizome weight of *A. lancea* in the AI treatment group was higher than that in the CK treatment group, but the result was not significant in this experiment. When barrier treatments were applied, the rhizome weight of *A. lancea* in the AN and AP treatment groups was similar to that of the CK treatment.

### Aboveground action of the intercropping system promoted the accumulation of volatile oils in *A. lancea*

The contents of active ingredients in plants represent an important indicator for evaluating the quality of TCMs (Zhang et al., 2022), and appropriate intercropping can improve their contents (Guo et al., 2020). The intercropping of *Mentha piperita* L. and *Vicia faba* L. has been demonstrated to increase the content of menthone in *Vicia faba* L. (Machiani et al., 2018), while the intercropping of *Dracocephalum moldavica (D. moldavica)* with *Glycine max* could increase the content of volatile oils in *D. moldavica* (Fallah et al., 2018). Similarly, by comparing the volatile oil contents of *A. lancea* between the AI and the CK treatment, the entire intercropping effect (aboveground + belowground) could promote the accumulation of volatile oils. By comparing the CK treatment with the AP treatment, we could verify our previous conjecture that the aboveground effect of maize can promote the accumulation of atractylon and atractylodin, but inhibit the accumulation of hinesol and β-eudesmol (Peng et al., 2021). It would be premature to draw the conclusion that part of the belowground effect lies in promoting or inhibiting the content of total volatile oils, so this needs further verification.

### Effects of *A. lancea* - maize intercropping on the physicochemical properties of the soil and the rhizosphere microbial community of *A. lancea*

Compared with the CK treatment, the rhizosphere microorganisms of intercropped plants may have both higher diversity and activity levels (Welbaum et al., 2010). Under intercropping conditions, there was a higher abundance of *Acidobacteria* bacteria (Zi et al., 2020). This finding is consistent with the existing literature. The relative abundance of the rhizosphere microorganism *Acidobacteria* in *A. lancea* under both the AI and AN treatment was higher than that in CK treatment (**Figures S1A, E**). Changing the soil microenvironment in the plant rhizosphere is known to be conducive to soil nutrient cycling (Kielak et al., 2016), thereby effectively improving the uptake of nitrogen (N), phosphorus (P), kalium (K), and other elements in the soil by plants, which is beneficial to their growth and development (Zeng et al., 2020; Wei et al., 2021). From the correlation analysis of **Figure 7A**, we observe that TP, TOC, Av.P, and Av.K were significantly positively correlated with the abundance of the majority of the different *A. lancea* rhizosphere microorganisms (the top 30 species), which was consistent with our **Figures 6A-C** analysis, where the relative abundance of these genera under the CK treatment was higher. The relative abundances of *Streptomyces*, *Candidatus Solibacter*, *Gemmatirosa*, and *Pseudolabrys* were lower in the CK treatment than in the AI treatment (**Figure S3**), and these microorganisms are known to form specific symbiotic relationships with plants to promote plant growth (Kong and Liu, 2022). From **Figure S2**, we found that these PGPR were most abundant following the AI treatment compared to the AN treatment, and compared with the AN and AP treatments, only the abundance of *Bradyrhizobium* was significantly higher after the AI treatment. This also provides an explanation for why some subterranean effects have failed to promote the growth of *A. lancea*.

## Conclusion

We analyzed the biomass, volatile oil concentration, physicochemical properties of the soil, and rhizosphere microbial community of *A. lancea* to characterize the underlying features of the root system interaction between *A. lancea* and maize. The results showed that in the *A. lancea* - maize intercropping system, maize could promote *A. lancea* to enrich the beneficial microorganisms in the rhizosphere, thereby promoting the growth of *A. lancea* as well as its accumulation of volatile oil. However, it is worth noting that when there is only partial rhizosphere interaction, the reported outcome may not be obvious or may even be entirely absent. In summation, we have demonstrated that intercropping maize can significantly change the soil physicochemical properties of *A. lancea*, thereby affecting the composition and structure of the microbial community in the rhizosphere of *A. lancea*, which proved beneficial to the enrichment of PGPR, and ultimately exerted a beneficial effect on the growth of *A. lancea*.

## Data availability statement

The raw sequence data reported in this paper have been deposited in the Genome Sequence Archive (Genomics, Proteomics & Bioinformatics 2021) in National Genomics Data Center (Nucleic Acids Res 2022), China National Center for Bioinformation / Beijing Institute of Genomics, Chinese Academy of Sciences. The data is publicly accessible at: https://ngdc.cncb.ac.cn/gsa, accession number CRA008493.

## Author contributions

All authors listed have contributed to the conception and design of this study. ZP and ZX: writing – original draft and data curation. LG, YZ, and LH: investigation, conceptualization, supervision, project administration, and funding acquisition. All authors have read and approved the final manuscript for publication.

## Funding

This research was funded by the following projects: The National Natural Science Foundation of China (No: 81874337, 81891014), the Scientific and Technological Innovation Project of the China Academy of Chinese Medical Sciences (CI2021A03903), Supported by the earmarked fund for CARS-21 (CARS-21) and Innovation Team and Talents Cultivation Program of National Administration of Traditional Chinese Medicine (ZYYCXTD-D-202005).

## Conflict of interest

Authors WX and LF were employed by Nanjing WaMing Agricultural Technology Co., Ltd.

The remaining authors declare that the research was conducted in the absence of any commercial or financial relationships that could be construed as a potential conflict of interest.

## Publisher’s note

All claims expressed in this article are solely those of the authors and do not necessarily represent those of their affiliated organizations, or those of the publisher, the editors and the reviewers. Any product that may be evaluated in this article, or claim that may be made by its manufacturer, is not guaranteed or endorsed by the publisher.

## Supplementary material

Supplementary Figure 1 Comparison of abundance in the top 10 represented phyla of the *A. lancea* rhizosphere microbial community under different root intercropping treatments employed for *A. lancea - maize* intercroping. The abundance comparison at the phylum level between the (A) CK and AN treatment, (B) CK and AP treatment, (C) AI and AN treatment, (D) AI and AP treatment, (E) CK and AI treatment, (F) AP and AN treatment. (* P<0.05; ** P<0.01; *** P<0.001).

Supplementary Figure 2 Comparison of abundance in the top 10 represented genera of the *A. lancea* rhizosphere microbial community under AI treatment, AN treatment, and AP treatment. Comparison of microbial abundance at the genus level between the (A) AI and AN treatment, (B) AI and AP treatment, (C) AP and AN treatment. (* P<0.05; ** P<0.01; *** P<0.001).

Supplementary Figure 3 Abundance comparison of the top 15 genera comprising the rhizosphere microbial community in *A. lancea* between the CK treatment and AI treatments in *A. lancea - maize* intercropping. (* *P*<0.05; ** *P*<0.01; *** P<0.001).

Supplementary Table 1 Spearman correlation analysis of soil physicochemical properties with growth and development and four volatile oils of *A.lancea*. FW, fresh weight; H, plant height; SD, stem diameter; BN, branch number; RW, rhizome weight; SN, sprout number; FR, fibrous root; Hin, hinesol; Eud, eudesmol; Atd, atractylodin; Atn, atractylon; TN, total nitrogen; TP, total phosphorus; TK, total kalium; TOC, total organic carbon; ${\text{NH}}_{4}^{+}$ , ammonium nitrogen; Av_P, available phosphorus; Av_K, available kalium.
