# Supplementary material for: Maize intercropping enriches plant growth-promoting rhizobacteria and promotes both the growth and volatile oil concentration of Atractylodes lancea
Source: Front Plant Sci. 2022 Oct 24;13:1029722. doi: 10.3389/fpls.2022.1029722 (PMC9638049; doi:10.3389/fpls.2022.1029722)
Supplement: Supplementary Figure 1 — Comparison of abundance in the top 10 represented phyla of the A. lancea rhizosphere microbial community under different root intercropping treatments employed for A. lancea - maize intercroping. The abundance comparison at the phylum level between the (A) CK and AN treatment, (B) CK and AP treatment, (C) AI and AN treatment, (D) AI and AP treatment, (E) CK and AI treatment, (F) AP and AN treatment. (* P<0.05; ** P<0.01; *** P<0.001). [file Presentation_1.pdf]

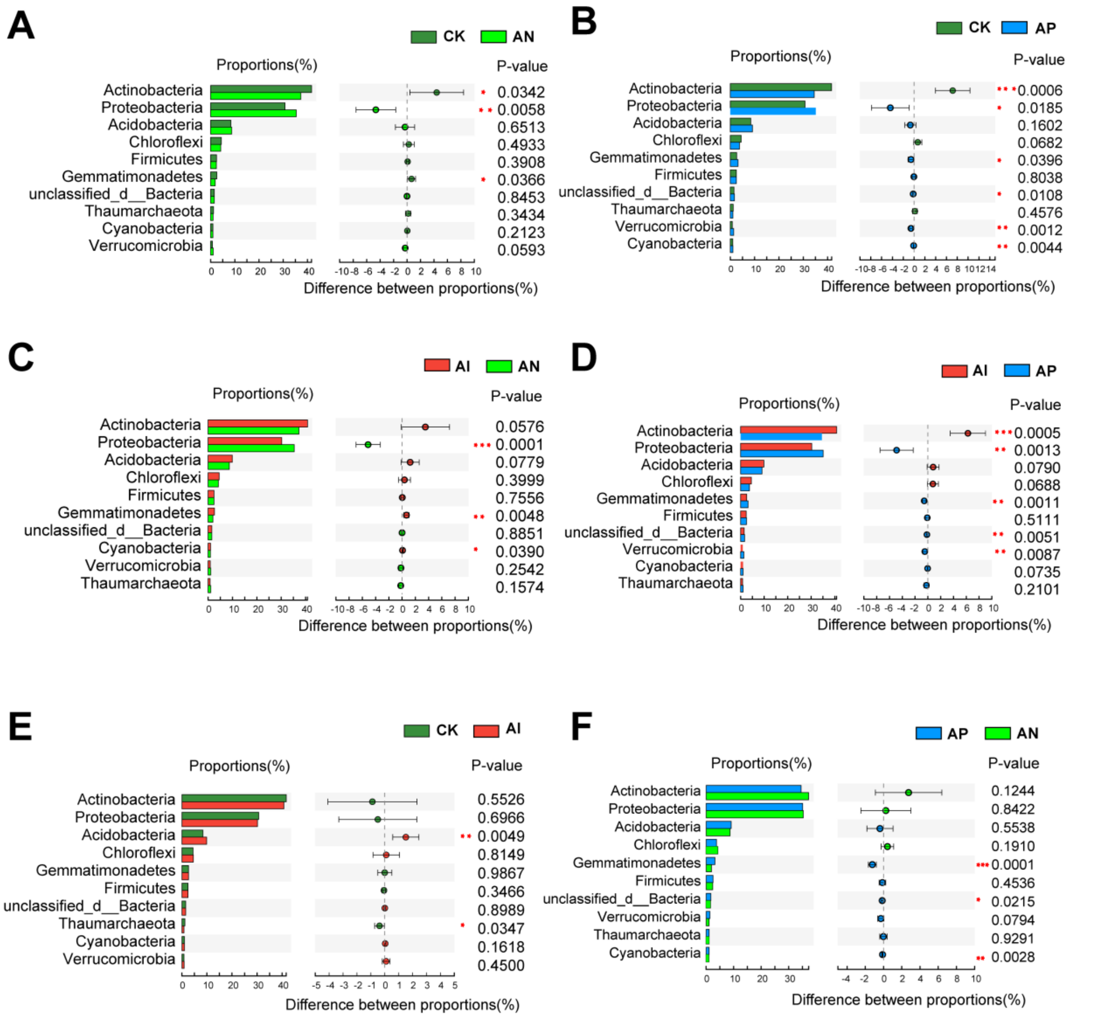

SUPPLEMENT FIGURE 1| Comparison of abundance in the top 10 represented phyla of the *A. lancea* rhizosphere microbial community under different root intercropping treatments employed for *A. lancea* - *maize* intercroping. The abundance comparison at the phylum level between the (A) CK and AN treatment, (B) CK and AP treatment, (C) AI and AN treatment, (D) AI and AP treatment, (E) CK and AI treatment, (F) AP and AN treatment. (* P<0.05; ** P<0.01; *** P<0.001).

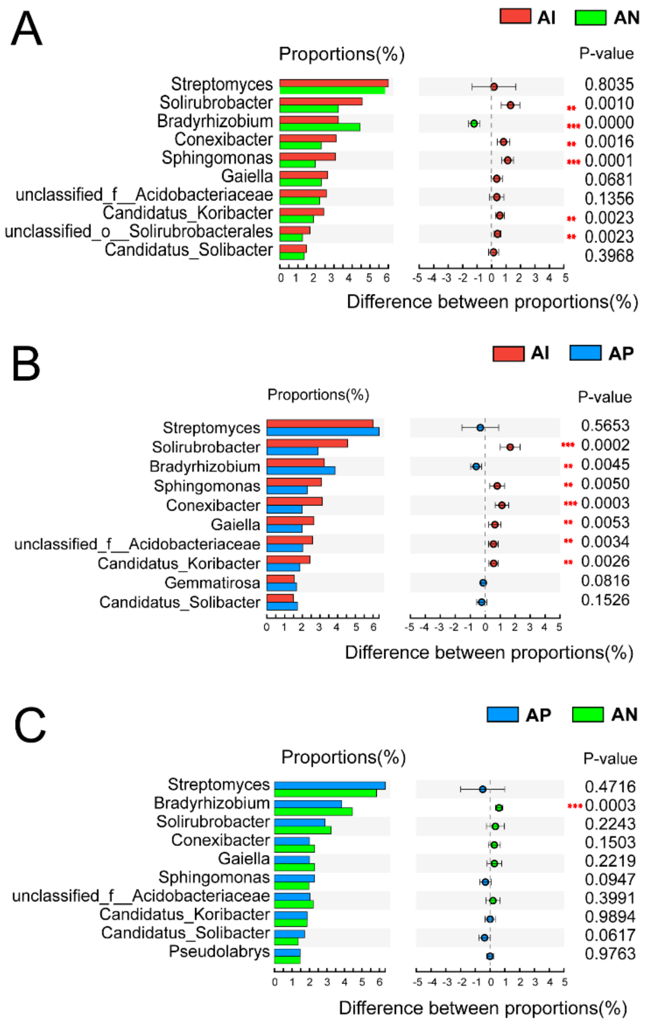

SUPPLEMENT FIGURE 2| Comparison of abundance in the top 10 represented genera of the *A. lancea* rhizosphere microbial community under AI treatment, AN treatment, and AP treatment. Comparison of microbial abundance at the genus level between the (A) AI and AN treatment, (B) AI and AP treatment, (C) AP and AN treatment. (* P<0.05; ** P<0.01; *** P<0.001).

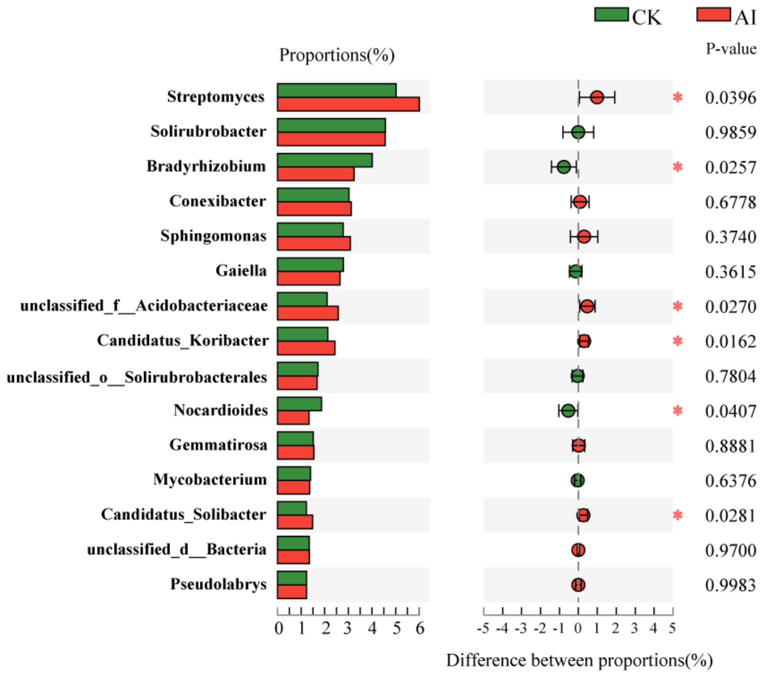

SUPPLEMENT FIGURE 3| Abundance comparison of the top 15 genera comprising the rhizosphere microbial community in *A. lancea* between the CK treatment and AI treatments in *A. lancea - maize* intercropping. (* *P*<0.05; ** *P*<0.01; *** P<0.001).

SUPPLEMENT Table 1| Spearman correlation analysis of soil physicochemical properties with growth and development and four volatile oils of *A.lancea*

	TN	TP	TK	TOC	NH_4^+	Av_P	Av_K	pH	C/N
**FW**	-0.214	0.315	0.131	-0.051	-0.253	0.174	-0.022	0.459*	-0.087
**H**	-0.204	0.138	0.232	-0.231	-0.308	0.046	-0.083	0.274	-0.015
**SD**	-0.15	0.216	-0.051	-0.135	-0.459*	-0.035	-0.01	0.392	0.001
**BN**	-0.219	0.215	-0.033	-0.129	-0.21	0.23	0.045	0.228	-0.133
**RW**	-0.21	0.308	0.097	-0.047	-0.233	0.193	0.005	0.399	-0.157
**SN**	-0.193	0.025	-0.346	-0.122	-0.507*	-0.193	0.011	0.357	0.09
**FR**	-0.314	-0.02	-0.259	-0.284	-0.386	-0.181	-0.241	0.478*	-0.081
**Hin**	-0.211	0.057	0.178	-0.191	-0.079	-0.011	-0.215	0.399	-0.108
**Eud**	-0.215	0.074	0.215	-0.178	-0.063	0.013	-0.219	0.423*	-0.074
**Atd**	-0.057	-0.34	-0.262	-0.105	-0.105	-0.221	-0.14	-0.128	-0.042

**Atn**	-0.126	-0.368	-0.285	-0.162	-0.099	-0.271	-0.183	-0.144	-0.056

the abbreviations: fresh weight (FW), plant height (H), stem diameter (SD), branch number (BN), rhizome weight (RW), sprout number (SN), fibrous root (FR), hinesol (Hin), eudesmol (Eud), atractylodin (Atd), atractylon (Atn). total nitrogen (TN), total phosphorus (TP), and total kalium (TK), total organic carbon (TOC)， ammonium nitrogen (NH_4^+), available phosphorus (Av_P), and available kalium (Av_K).
